# Atorvastatin Downregulates *In Vitro* Methyl Methanesulfonate and Cyclophosphamide Alkylation-Mediated Cellular and DNA Injuries

**DOI:** 10.1155/2018/7820890

**Published:** 2018-04-03

**Authors:** Carlos F. Araujo-Lima, Larissa S. A. Christoni, Graça Justo, Maria N. C. Soeiro, Claudia A. F. Aiub, Israel Felzenszwalb

**Affiliations:** ^1^Department of Biophysics and Biometry, Rio de Janeiro State University (UERJ), Rio de Janeiro, RJ, Brazil; ^2^Department of Genetics and Molecular Biology, Rio de Janeiro State Federal University (UNIRIO), Rio de Janeiro, RJ, Brazil; ^3^Laboratory of Cellular Biology, Oswaldo Cruz Institute (FIOCRUZ/IOC), Rio de Janeiro, RJ, Brazil; ^4^Department of Biochemistry, Rio de Janeiro State University (UERJ), Rio de Janeiro, RJ, Brazil

## Abstract

Statins are 3-hydroxy-3-methylglutaryl-coenzyme A (HMG-CoA) reductase inhibitors, and this class of drugs has been studied as protective agents against DNA damages. Alkylating agents (AAs) are able to induce alkylation in macromolecules, causing DNA damage, as DNA methylation. Our objective was to evaluate atorvastatin (AVA) antimutagenic, cytoprotective, and antigenotoxic potentials against DNA lesions caused by AA. AVA chemopreventive ability was evaluated using antimutagenicity assays (*Salmonella*/microsome assay), cytotoxicity, cell cycle, and genotoxicity assays in HepG2 cells. The cells were cotreated with AVA and the AA methyl methanesulfonate (MMS) or cyclophosphamide (CPA). Our datum showed that AVA reduces the alkylation-mediated DNA damage in different *in vitro* experimental models. Cytoprotection of AVA at low doses (0.1–1.0 *μ*M) was observed after 24 h of cotreatment with MMS or CPA at their LC_50_, causing an increase in HepG2 survival rates. After all, AVA at 10 *μ*M and 25 *μ*M had decreased effect in micronucleus formation in HepG2 cells and restored cell cycle alterations induced by MMS and CPA. This study supports the hypothesis that statins can be chemopreventive agents, acting as antimutagenic, antigenotoxic, and cytoprotective components, specifically against alkylating agents of DNA.

## 1. Introduction

Alkylating agents (AAs), at the widest sense, are compounds able to substitute a hydrogen atom in other molecules by an alkyl radical, involving electrophilic attack by the AA. The definition is extended to the reactions involving addition of the radical to a molecule containing an atom in a lower valence state, as the sulfonates [1]. These agents that induce DNA methylation can act through covalent modification of DNA to generate mismatching base derivatives and lesions that interrupts genetic replication [2].

Statins are drugs largely used to inhibit cholesterol synthesis by blockage of HMG-CoA reductase [3]. Statin pleiotropic effects are the nonhypocholesterolemic-related new roles that this class of drugs presents [4]. In eukaryotic cells, the antineoplastic effect of statins occurs by suppression of mevalonate biosynthesis, a precursor of important isoprenoid intermediates which are added during posttranslational modification of a variety of proteins such as subunits Ras and Rho of small G protein [5]. These modifications in Rho GTPases can induce actin cytoarchitectonic rearrangement by reducing the focal adhesion regions, stress fiber formation, and cell pseudopod emission, disfavoring cellular migration and phagocytosis [6]. In this sense, our intent was to observe possible chemopreventive effects of the compounds on different biological models exposed to chemical injury induced by AA.

## 2. Materials and Methods

### 2.1. Compounds

For antimutagenesis and cytoprotection assays, AVA (CAS #134523-00-5) and the AA (methyl methanesulfonate (MMS; CAS #66-27-3), cyclophosphamide (CPA; CAS #50-18-0)) stock solutions were prepared in dimethyl sulfoxide (DMSO) with the final concentrations of the solvent never exceeding 1.0%, which did not exert any toxicity (data not shown), and aliquots were stored at −20°C.

### 2.2. Scavenging of 2,2-Diphenyl-1-picrylhydrazyl (DPPH) Assay

The free radical scavenging activity was measured by following microplate procedures as previously described [7]. One hundred microliters of the sample dilutions with five concentration levels (varying from 0 to 2000 *μ*M in DMSO) was added to two identical groups of wells in a 96-well microplate. The same volume of 0.1 mM DPPH-methanol solution was added to each well of one group (samples), and methanol (100 mL) was added to the other group (blanks). The control was prepared by mixing the DPPH-methanol solution with the sample solvent or butylated hydroxytoluene (BHT). The solutions were mixed thoroughly, covered, and allowed to react in the dark at room temperature for 40 min. The absorbance was measured at 517 nm using a microplate reader (Quant, BioTek Instruments Inc.), and the scavenging activity was calculated from the absorbance values according to the following equation: % scavenging = (control sample)/(control blank) × 100%. The antioxidant properties of the samples were expressed as half the maximal effective concentrations (EC_50_) obtained by interpolation from the linear regression analysis. BHT was used as the positive control.

### 2.3. Biological Models

#### 2.3.1. Bacteria

*Salmonella enterica* serovar *typhimurium (S. typhimurium)* strains TA100, TA1535, TA104, and TA102 from the authors' laboratory stock were used as described by Maron and Ames [8] in the antimutagenicity assay.

#### 2.3.2. Cell Culture

Human hepatocellular carcinoma cells (HepG2) obtained from the American Type Culture Collection (Manassas, VA) were cultured in a minimum Eagle's medium (MEM, Gibco®, USA) containing 10% fetal bovine serum (FBS) plus 100 *μ*g/mL streptomycin and 100 *μ*g/mL penicillin at 37°C in a 5% CO_2_ atmosphere. Logarithmic-phase cells were used in all the experiments [9].

### 2.4. Antimutagenicity in a Bacterial Model

We carried out the coexposure protocol of the antimutagenicity assay to investigate the potential of the compound to protect against alkylation-mediated genetic mutation in *S. typhimurium* TA100, TA102, TA104, and TA1535 strains according to Ajith and Soja [10]. The test proceeded both in the absence and presence of a metabolic activation system (4% S9 mix, Aroclor preinduced, from MOLTOX Inc., USA). DMSO 1% served as the negative control. For the assays without metabolic activation, 0.5 mL of a 0.1 mol/L sodium phosphate buffer (pH 7.4) was added, and for the assays in the presence of metabolic activation, 0.5 mL of S9 mix was mixed with a 0.1 mL culture medium (2 × 10^8^ cells/mL) plus 0.1 mL of AVA solutions (0–1000 *μ*M) and 0.1 mL MMS (100 *μ*g/plate) in the absence of metabolic activation and CPA (100 *μ*g/plate) in metabolic active conditions. The mixtures were incubated in a shaker at 37°C (preincubation) under light protection. After a total of 60 min of cotreatment, the mixtures were added to and mixed with 2 mL top agar containing 0.05 mmol/L L-histidine and D-biotin for the *S. typhimurium* strains. Each of these was then spread on a minimal glucose agar (1.5% agar, Vogel-Bonner medium E, containing 2% glucose) plate. After the top agar solidified, the plates were incubated at 37°C for 60–72 h. Each tester strain was assayed in triplicate and repeated at least twice, and the number of revertant colonies was counted for each tester strain and treatment group [11]. The counts of revertant colonies were obtained to build a dose-response curve and calculate the percentage of reduction. Statistical differences between the groups were analyzed by a one-way ANOVA (*p* < 0.05) and Tukey's post hoc test.

When we did not detect a significant reduction in cotreatment, we carried out the pretreatment and posttreatment protocols, according to our previous study [12]. In the pretreatment protocol, the bacterial suspensions were incubated in a buffer or S9 mix with AVA for 30 minutes. After this period, the mutagen (MMS in −S9 condition and CPA in +S9 condition) was added and the mixtures were incubated for 30 minutes. The posttreatment protocol consisted in the incubation of the bacterial suspension with the mutagen for 30 minutes, and after the addition of AVA, the mixtures were incubated for 30 minutes more. The % of reduction was determined by linear regression considering 0% the background count and 100% the group exposed only to MMS or CPA.

To determine the cytotoxic effect, after 60 min incubation, the assay mixtures were diluted in 0.9% NaCl (*w*/*v*) to obtain a suspension containing 2 × 10^2^ cells/mL. A suitable aliquot (100 *μ*L) of this suspension was plated on nutrient agar (0.8% bacto nutrient broth (Difco), 0.5% NaCl, and 1.5% agar). The plates were then incubated at 37°C for 24 h, and the colony-forming units (CFU) were counted to obtain the percentage of survival. All the experiments were done in triplicate and were repeated at least twice. Statistical differences between the groups were analyzed by a one-way ANOVA (*p* < 0.05) and Tukey's post hoc test [12].

### 2.5. Cytoprotective Assay of HepG2 Cells

Fresh HepG2 cells were seeded at a density of 1 × 10^5^/well. The water-soluble tetrazolium salt assay (WST-1) (4-[3-(4-iodophenyl)-2-(4-nitrophenyl)-2H-5-tetrazolio]-1,3-benzene disulfonate) (Roche Co., South San Francisco, CA) was used to determine the number of viable cells after 24 h of exposure to AVA and the AAs (0 to 1000 *μ*M. Briefly, after treatment, the culture medium was replaced by a 90 *μ*L fresh culture medium and a 10 *μ*L WST-1 reagent and incubated at 37°C and 5% CO_2_ for 2 h. The absorbance was then measured at 440 nm according to the kit protocol and according to Ferraz et al. [13]. The intensity of the yellow color in the negative control (DMSO 1%) wells was designated as 100% viability, and all further comparisons were based upon this reference level to determine the lethal concentration (LC_50_) to 50% of cultured cells.

After the determination of LC_50_ of AVA, MMS, and CPA, fresh HepG2 cells were seeded at a density of 1 × 10^5^/well and were coincubated with each AA at its LC_50_ and AVA (from 0 to 100 *μ*M) for its cytoprotective capacity evaluation. After 24 h of coexposure, the culture medium was replaced by a 90 *μ*L fresh culture medium and 10 *μ*L WST-1 and incubated at 37°C and 5% CO_2_ for 2 h. The absorbance was then measured following the protocol as described before. The survival rates were determined in comparison to the negative control. Statistical differences between the groups were analyzed by a one-way ANOVA (*p* < 0.05 to <0.001) and Tukey's post hoc test.

### 2.6. Micronuclei in HepG2 Cells

The HepG2 cells were seeded at a density of 1 × 10^5^/mL into 24-well plates (1 mL/well). The CPA at 60 *μ*M or MMS at 0.5 *μ*M was coincubated with AVA at 10 *μ*M and 25 *μ*M or incubated alone for 6 h or 24 h. DMSO 1% was used as the negative control. After exposure to the compounds, the cells were incubated for 24 h more and the cytogenetic studies were carried out in triplicate and *N* = 3 [14]. In order to determine the mitotic index and the number of cells with micronuclei, the medium was replaced by a cold methanol-glacial acetic acid (3 : 1) fixative for 30 min and the cells were then rinsed with distilled water for 2 min and air dried. The fixed cells were stained with 4,6-diamidino-2-phenylindole (DAPI) (0.2 pg/mL), dissolved in a McIlvaine buffer (0.1 M citric acid plus 0.2 M Na_2_HPO_4_, pH 7.0) for 60 min, washed with a McIlvaine buffer for 5 min, briefly rinsed with _dd_H_2_O, and mounted in glycerol. To determine the mitotic index and the number of cells with micronuclei, 2000 cells per well (6000 cells per concentration) were analyzed using fluorescence microscopy (Reichert Univar) with an excitation wavelength of 350 nm. The results for micronuclei were presented as the percentage of cells containing micronuclei in 6000 cells/concentration. Statistical differences between the groups were analyzed by a one-way ANOVA (*p* < 0.01) and Tukey's post hoc test.

### 2.7. Flow Cytometry Cell Cycle Analysis

The cells (1 × 10^5^) were washed in PBS solution and centrifuged at 400 ×g for 5 min, and after, the cells were suspended in DNA staining solution (0.3% Triton X-100 and 50 *μ*g/mL propidium iodide (PI) in a 43 mM citrate buffer), as previously described. After 45 minutes of treatment with 50 *μ*g/mL ribonuclease A (Sigma, EUA), the PI fluorescence was determined (10,000 events per sample) in a Gallios flow cytometer (Beckman Coulter, USA). Data were analyzed by the Summit v4.3 software. The experiments were done at least three times, and statistical analysis was performed by one-way ANOVA followed by a Tukey's post hoc test [15].

## 3. Results

### 3.1. DPPH Assay

**Figure 1:**
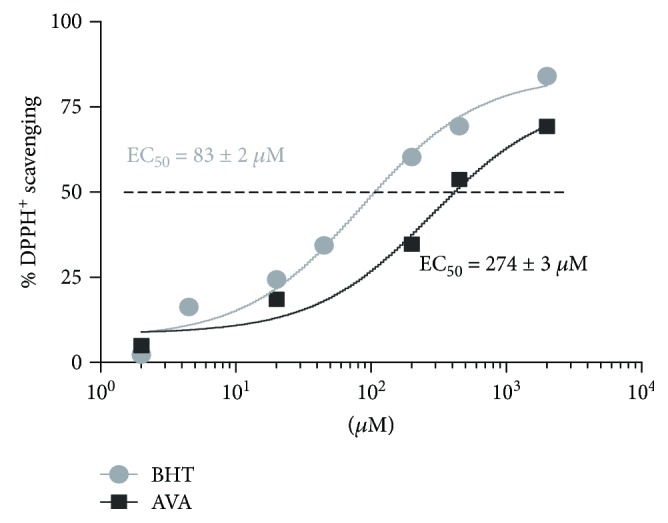
Atorvastatin (AVA) direct antioxidant activity by a DPPH^+^ scavenging model. After 50 minutes of incubation with DPPH^+^ free radicals, AVA scavenging potential was measured by spectrophotometry at 517 nm. A clear Q-curve (*R*^2^ = 0.9733) can be evidenced, representing a dose-response phenomenon, and AVA's EC_50_ was 274 ± 3 *μ*M. Butylated hydroxytoluene (BHT) was used as an antioxidant (positive control) and presented as *R*^2^ = 0.9712 and EC_50_ = 83 ± 2 *μ*M. The experiments were done in triplicate and repeated 3 times (*n* = 3).

After incubation with DPPH^+^, AVA was capable to exert DPPH free radicals scavenging dose-dependently (Figure 1). AVA obtained an EC_50_ = 274 ± 3 *μ*M and showed itself to be a good direct antioxidant, with similar results to the positive control, even though BHT's EC_50_ was lower (83 ± 2 *μ*M).

### 3.2. Antimutagenicity in a Bacterial Model

In the antimutagenicity evaluation using a bacterial model, AVA presented no cytotoxic effect to *S. enterica* serovar *typhimurium*-tested strains, both in the presence and absence of exogenous metabolic activation (data not shown).

After the cotreatment with alkylating agents, AVA presented dose-dependent antimutagenic effects against MMS-directed DNA damage for TA104 (32.3% and 55.7% of reduction at 200 *μ*M and 1000 *μ*M, resp.), TA102 (29% and 41.2% of reduction at 200 *μ*M and 1000 *μ*M) (Table 1), and TA1535 (from 45.7% to 91.3% of reduction in all the tested concentrations) (Table 2). AVA also presented antimutagenicity against CYP metabolism-dependent DNA injury caused by CPA for TA104 (25%, 29%, and 41% of reduction, respectively, at 100 *μ*M, 200 *μ*M, and 1000 *μ*M), TA102 (30% of reduction at 1000 *μ*M) (Table 1), and TA1535 (30.6% and 42.2% of reduction at 200 *μ*M and 1000 *μ*M, resp.) (Table 2). At last, AVA did not protect TA100 (Table 2) against MMS nor CPA mutagenicity.

Due to this lack of chemopreventive effects just on TA100, we carried out pretreatment and posttreatment protocols using this strain (Table 3). AVA exerted antimutagenic activity to TA100 on all of the pretreated concentrations both in the presence and absence of metabolic conditions, presenting the highest percentages of reduction of revertants. Notwithstanding, posttreatments with AVA concentrations were not able to reduce the DNA injuries caused directly by MMS or those related to the metabolism of CPA.

### 3.3. Cytoprotection of HepG2 Cells

**Figure 2:**
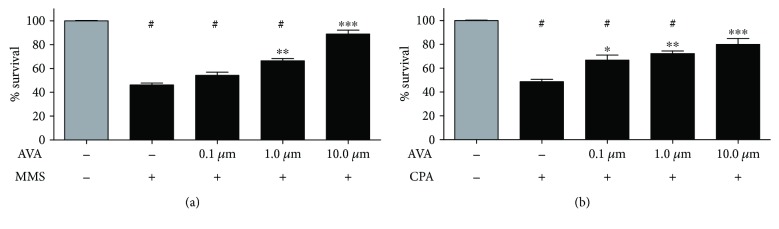
Effect of cotreatment with atorvastatin (AVA) after 24 h of coexposure with alkylating agents. HepG2 cells were coexposed to AVA from 0.1 to 100 *μ*M. It is possible to observe that AVA induced a significant cytoprotective effect in hepatic cells coexposed to (a) 20 *μ*M MMS at 1.0 and 10.0 *μ*M. The same effect was observed against (b) 100 *μ*M CPA from 0.1 to 10.0 *μ*M (^#^*p* > 0.001 versus the negative control and ^∗^*p* > 0.05; ^∗∗^*p* > 0.01; ^∗∗∗^*p* > 0.001 versus CPA or MMS only; *n* = 4 in triplicate; one-way ANOVA followed by a Tukey's post hoc test).

The hepatotoxicity of the compounds using HepG2 cells at 24 h of exposure is presented in Table 4. AVA showed LC_50_ > 1000 *μ*M. The AA presented different grades of hepatotoxicity. CPA's LC_50_ was 98.71 ± 11.50 *μ*M. MMS was more hepatotoxic, presenting LC_50_ = 18.67 ± 6.67 *μ*M. Using the alkylating agent concentrations around the LC_50_ to evaluate the AVA cytoprotective effects, which means that there is the potential to reduce cell death induced by the DNA AA in our specific case, it is possible to observe that AVA induced a significant protection in hepatic cells coexposed to MMS at 1.0 and 10.0 *μ*M (Figure 2(a)). The same effect was observed against CPA (Figure 2(b)) from 0.1 to 10.0 *μ*M.

### 3.4. Micronuclei in HepG2 Cells

**Figure 3:**
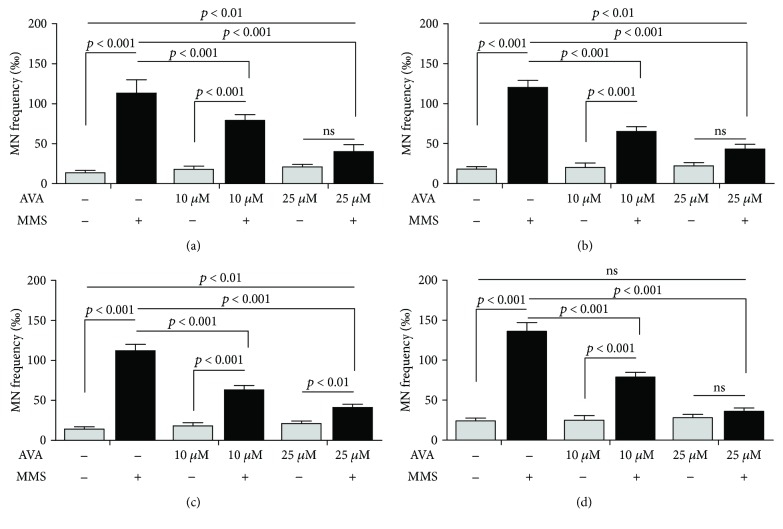
Effect of cotreatment with atorvastatin (AVA) on methyl methanesulfonate- (MMS-) or cyclophosphamide- (CPA-) induced micronuclei in HepG2 cells. HepG2 cells were coincubated with AVA at 10 and 25 *μ*M with10 *μ*M MMS after (a) 6 h or (b) 24 h of exposure. The coincubation with 60 *μ*M CPA during (c) 6 h or (d) 24 h followed the same protocol. 2000 cells were scored per treatment for each experiment (*n* = 3 in triplicate; one-way ANOVA followed by a Tukey's post hoc test).

Figure 3 shows the micronucleated HepG2 cell counts of coexposure to AVA and 10.0 *μ*M MMS after 6 h (Figure 3(a)) and 24 h (Figure 3(b)). After exposure to MMS, it is possible to observe a significant decrease in micronucleus formation in coincubated cells to AVA at 6 and 24 h, from 6-7 fold (in only MMS-exposed cells) to 3-4 fold and 1-2 fold in comparison to the negative control at 10.0 *μ*M or 25.0 *μ*M, respectively. After 6 h (Figure 3(c)) and 24 h (Figure 3(d)) of coexposure to 60.0 *μ*M CPA, AVA showed the same behavior, decreasing the fold from 5-6 fold to 3-4 fold and 1-2 fold in comparison to the negative control at 10.0 *μ*M or 25.0 *μ*M.

### 3.5. Cell Cycle Analysis

**Figure 4:**
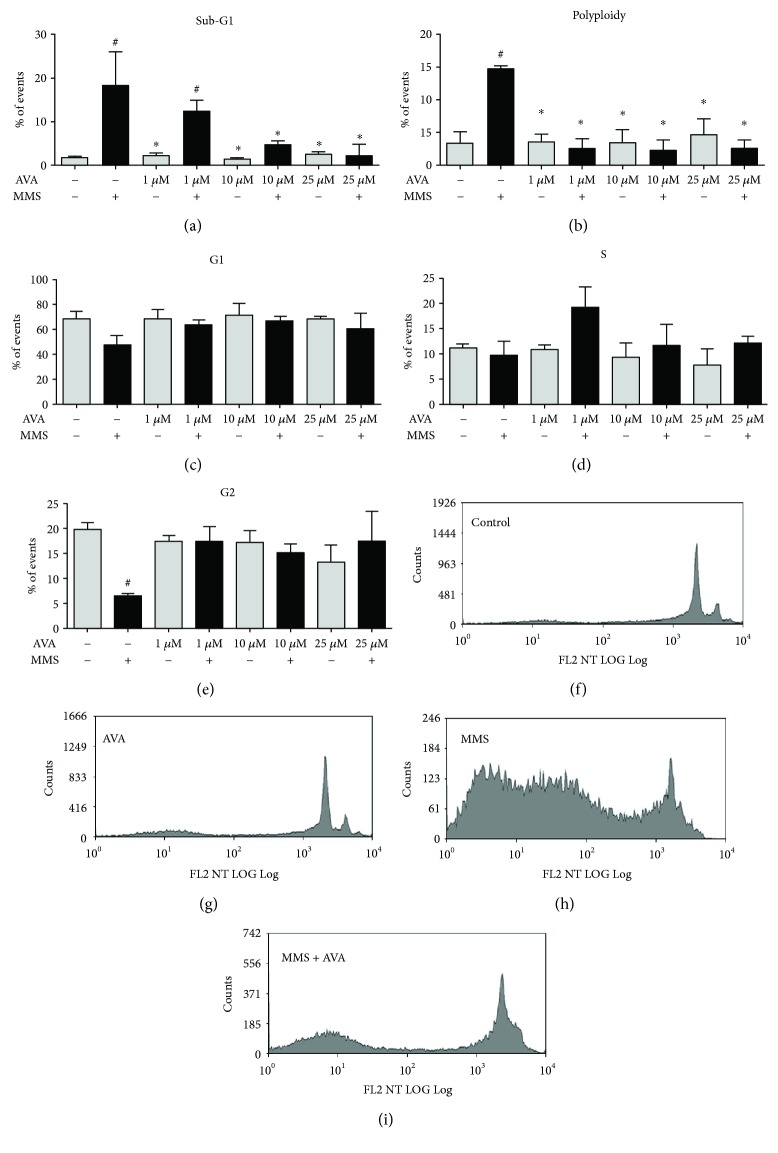
Cell cycle analysis of HepG2 cells after treatment with atorvastatin (AVA) and also cotreatments with AVA and methyl methanesulfonate (MMS). HepG2 cells were incubated with 1, 10, and 25 *μ*M AVA or 20 *μ*M MMS and also coincubated with 1, 10, and 25 *μ*M AVA plus 20 *μ*M MMS during 24 h. The negative control was DMSO 1%. The histograms represent the percentages of cell cycle phases in each condition by flow cytometry. Data of 104 cells were analyzed using the Summit v4.3 software (Dako Colorado Inc., USA). Cotreatment with AVA reduced the sub-G1 percentage of cells in a dose-dependent manner (a) and polyploid cells (b), in comparison to only MMS-exposed cells, without affecting G1 (c) and S (d) phases and restored the number of G2 cells (e). The representative histograms demonstrated that in comparison to the control (f), 25 *μ*M AVA (g) did not induce alterations on the cell cycle pattern. On the other hand, 20 *μ*M MMS (h) induced several modifications on the cell cycle pattern, but the cotreatment with 25 *μ*M AVA (i) in MMS-exposed cells restored the cell cycle pattern (*n* = 3; ^#^*p* > 0.001 versus the control and ^∗^*p* > 0.001 versus MMS only; one-way ANOVA followed by a Tukey's post hoc test).

We observed that after exposure to MMS, HepG2 cell subsets at different stages of the cell cycle were significantly different from what was observed in the unexposed control (Figure 4). AVA reduced the sub-G1 percentage of cells (Figure 4(a)) in a dose-dependent manner, from 19% in untreated cells to 12%, 4%, and 2% in its cotreatment at 1 *μ*M, 10 *μ*M, and 25 *μ*M, respectively. AVA also reduced the polyploid subpopulation (Figure 4(b)), from 15% after exposure just to MMS to the background counts (3-4%) in cotreatment. AVA and MMS cotreatment did not affect G1 (Figure 4(c)) and S (Figure 4(d)) phases and restored the number of cells in the G2 phase (Figure 4(e)) that was reduced in only MMS-exposed cells. The representative histograms demonstrated that, in comparison to the control (Figure 4(f)), 25 *μ*M AVA (Figure 4(g)) did not induce alterations on the cell cycle pattern. On the other hand, 20 *μ*M MMS (Figure 4(h)) induced several modifications on the cell cycle pattern, but the cotreatment with 25 *μ*M AVA (Figure 4(i)) in MMS-exposed cells restored the cell cycle pattern.

**Figure 5:**
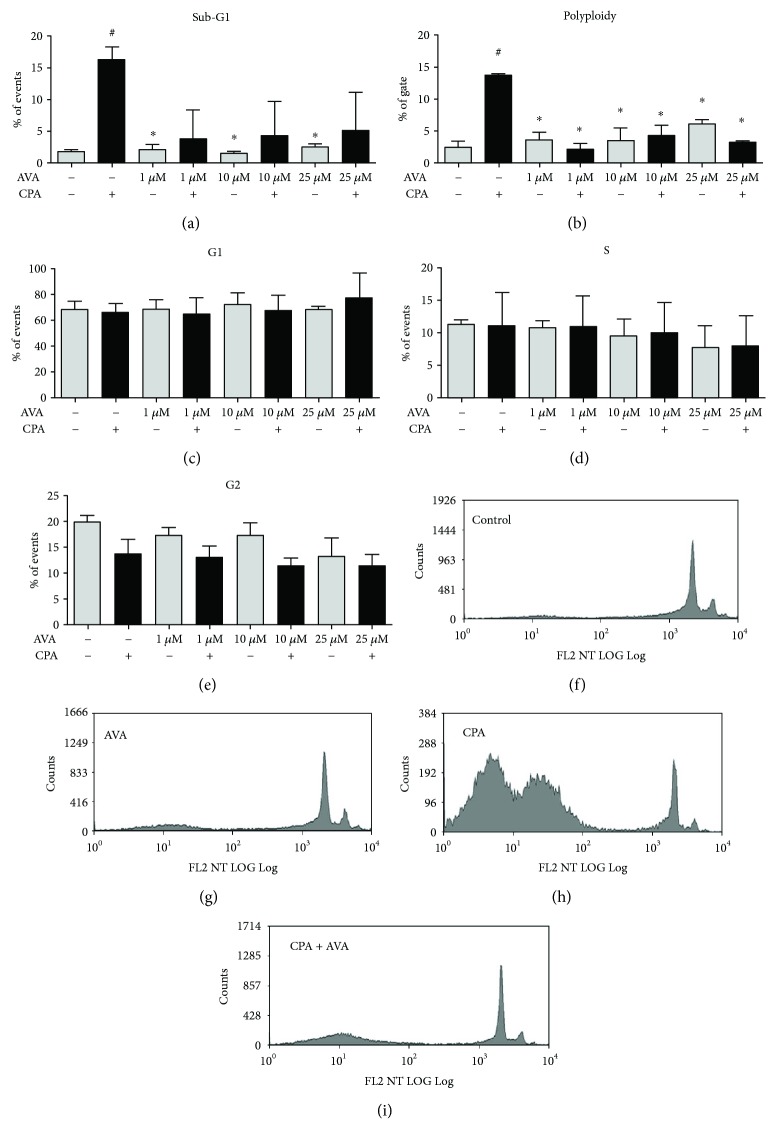
Cell cycle analysis of HepG2 cells after treatment with atorvastatin (AVA) and also cotreatments with AVA and cyclophosphamide (CPA). HepG2 cells were incubated with 1, 10, and 25 *μ*M AVA or 60 *μ*M CPA or coincubated with 1, 10, and 25 *μ*M AVA plus 20 *μ*M CPA during 24 h. The negative control was DMSO 1%. The histograms represent the percentages of cell cycle phases in each condition by flow cytometry. Data of 104 cells were analyzed using the Summit v4.3 software (Dako Colorado Inc., USA). Cotreatment with AVA reduced the sub-G1 percentage of cells (a) and polyploid cells (b), in comparison to only CPA-exposed cells, without affecting G1 (c), S (d), and G2 (e) phases. The representative histograms demonstrated that in comparison to the control (f), 25 *μ*M AVA (g) did not induce alterations on the cell cycle pattern. On the other hand, 60 *μ*M CPA (h) increased sub-G1 percentage of cells, but after the cotreatment with 25 *μ*M AVA (i) in CPA-exposed cells, it was restored (*n* = 3; ^#^*p* > 0.001 versus the control and ^∗^*p* > 0.001 versus CPA only; one-way ANOVA followed by a Tukey's post hoc test).

The same behavior was observed after exposure to CPA with HepG2 cell subsets at different stages of the cell cycle presenting significantly different counts from what was observed in the unexposed control (Figure 5). AVA also reduced the sub-G1 percentage of cells (Figure 5(a)), from 17% in untreated cells to the background counts (3-4%) that did not exert dose dependence. AVA also reduced the polyploid subpopulation (Figure 5(b)) from 13% after exposure just to CPA to the background counts (3–5%) in cotreatment; besides, the incubations with different AVA treatments increased the number of polyploidy cells, even though there is no significance. AVA and CPA cotreatment did not affect G1 (Figure 4(c)), S (Figure 4(d)), and G2 phases (Figure 4(e)). The representative histograms demonstrated that, in comparison to the control (Figure 5(f)), 25 *μ*M AVA (Figure 5(g)) did not induce alterations on the cell cycle pattern. On the other hand, 20 *μ*M MMS (Figure 5(h)) induced several modifications on the cell cycle pattern, but the cotreatment with 25 *μ*M AVA (Figure 5(i)) in MMS-exposed cells restored the cell cycle pattern.

## 4. Discussion

According to the study of Ajith and Soja [10], atorvastatin (AVA) and lovastatin (LOVA) were able to exert chemopreventive effects against direct mutagens in a bacterial reverse mutation model using *Salmonella enterica* serovar *typhimurium* TA98 and TA100 strains in the absence of metabolic activation. The antimutagenic effects of AVA and LOVA against the direct mutagens sodium azide or 4-nitro-*o*-phenylenediamine in a bacterial reverse mutation model using *Salmonella enterica* serovar *typhimurium* TA98 and TA100 strains were described previously. AVA significantly inhibited the mutagenic response, which was evident by the decrease in revertant colony counts in cotreated plates [10].

In our study, we used four *Salmonella enterica typhimurium* strains to be able to detect DNA damage caused by base-pair substitution/transition. Our results corroborate the Ajith and Soja study [16], once AVA showed itself being more protective against direct than indirect induction in a bacterial model. Mutagenesis is not a passive process, and the modifications in DNA sequence can be mediated by mechanisms of repair [16]. This active and multifactorial process of DNA modifications based on DNA impairment and repair is named genomic instability [17]. TA1535 and TA104, strains that are deficient in error-prone recombination repair (REC), were more effective than the REC-proficient correspondent strains (TA100 and TA102, resp.) in exerting chemoprevention against AA damage. These REC-proficient variants can produce an endonuclease mediated by *RecA* SOS response, which could play a role in “nick and gap” formation in the mutagenized DNA [18]. Besides this, TA100 and TA102 can activate DNA repair mediated by an error-prone polymerase [19].

In relation to TA1535/TA100 (TA1535, *pKM101^+^*), these strains are capable to detect mutations by substitution of G:C to A:T pairs in GGG sites of hotspot locus *hisG46*. They can detect primary DNA modifications, after a replication cycle, as alkylation in purines, mainly in guanine, as N-(2-chloroethyl)-N-[2-(7-guaninyl)ethyl]amine or an hydroxylated mustard arm (N-(2-hydroxyethyl)-N-[2-(7-guaninyl)ethyl]amine) [20], the kind of damage induced by CPA and O^6^-alkyl-G formation and induced by MMS [21]. The protective effect was more evident against MMS because this mutagen acts predominantly by alkylating guanines and favoring adduct formation [22]. In relation to TA104 and TA102 (TA 104, *pKM101^+^*), both strains are capable to detect thymine alkylation by formation of O^4^-alkyl-T due to A:T to G:C transition and mismatch recognizing [20–22], and AVA was more antimutagenic to TA104 than to TA102. Specifically in this case, AVA was protective to TA1535 and was not to TA100 in coincubation, which means that probably REC has an important role in AVA antimutagenesis, and also, base excision repair (BER) can play a primordial role in this process.

According to De Flora et al. [11], the implementation of protocols that include pre- and posttreatments are scientifically relevant because it allows predicting some aspects about the mechanism of action (MoA) in antimutagenesis assays. In general, the literature recommends to perform cotreatment protocol as a trial model, once the most part of antimutagens can demonstrate some protection in combined exposure, and then perform pre/posttreatments after, to obtain more mechanistic information. Antimutagenicity's MoA in cotreatment is related to general antimutagenic activity and also can be related to membrane responses. If a compound just exerts antimutagenic effect on pretreatment, the MoA is related to extracellular events as an interruption of promutagen shift, free radical scavenging capacity or other antioxidative property. Withal, if a compound is antimutagenic just on posttreatment, it means that this MoA is related to this compound ability to reduce the DNA attachment of the mutagen or activation of repair mechanisms and/or induction of DNA dismutation [23]. In this sense, the antimutagenic activity observed for TA100 just in pretreatment suggests that AVA can exert directly free radical scavenging, which is in accord with our DPPH model results.

Rossini et al. [24] demonstrated that the most frequent *TP53* mutations in esophageal cancer varies according to the injury that the tissue was exposed. The frequency of G:C to A:T CpG or non-CpG mutations in TP53 was higher in patients exposed to inflammatory injuries. In our model, the antimutagenic effect of AVA was more relevant on *Salmonella* strains that detect G:C to A:T substitution which corroborate the hypothesis that the chemopreventive effects of AVA are mediated by downregulation of the redox status, reducing the genomic instability.

In eukaryotic cells, statins can contribute to oxidative stress modulation in different tissues. AVA was able to enhance glutamate via glutamate synthase activity in hippocampal neural cells after hypoxia and starvation conditions [25]. Comparatively, cells treated with AVA produced less ROS than the untreated cells. In the same sense, LOVA were capable to prevent genotoxic and cytotoxic effects caused by doxorubicin, etoposide, and MMS in human umbilical vein endothelial cells (HUVEC) by reduction of FASr, procaspase 2, and phosphorylated JNK-1 [26].

On the other hand, Gajski et al. [27] observed AVA-mediated genotoxic damage in human lymphocyte chromosome aberrations, sister chromatid exchange and increasing in tail length and intensity in lymphocyte comet assay even at nM concentrations. According to the authors, this DNA damage was caused by oxidative stress, observed in Fpg-modified comet assay. These evidences go against the original study about the AVA's safety profile that demonstrated in a complete toxicological screening that AVA is a safe drug [28]. Reis et al. also showed LOVA's capacity to enhance heme oxygenase 1 and reduction of lipid peroxidation in cerebral tissues [29]. AVA also induced antioxidative effect and reduced pathophysiological impairments mediated by host immunity in malaria infection [30].

The preantineoplastic effect of statins occurs by suppression of mevalonate biosynthesis, a precursor of important isoprenoid intermediates which are added during posttranslational modification of a variety of proteins such as subunits Ras and Rho of small G protein. These proteins are involved in cell cycle progression, cell signaling, and membrane integrity. The inhibition of Rho activation reversed the metastatic phenotype of human melanoma cells [5].

Jialal et al. [31] demonstrated a reduction in reactive protein C and hepatic acute phase proteins after treatment with statins in a follow-up clinical trial, suggesting that possibly these drugs can act in hepatic oxidative damage chemoprevention. Our results go in the same way of this evidence, showing an AVA capacity to reduce HepG2 cell death in coexposure to different AAs. On the micronucleus assay, we choose the AA concentration based on using noncytotoxic doses (a concentration lower than LC_50_) and it was possible to observe that AVA presented a dose-response antigenotoxic effect against the AAs. In addition, against the nonmetabolism-dependent AA (MMS), AVA reduced the frequency in damaged cells earlier at the lower concentration, reaching the level of micronucleated cells to the same range of the negative control at 6 h. Against the metabolism-dependent AA (CPA), AVA just reached the level of micronucleated cells to the range of the negative control after 24 h of coexposure, displaying a late response.

At last, the cell cycle analysis by the flow cytometry approach allowed us to confirm the cytoprotective aspects that were observed by the other methodologies. Exposing HepG2 cells to the same AA concentration that we used on micronucleus assay and co-incubating the cells with AA and AVA treatments, we observed a reduction on Sub-G1 subpopulations, in comparison to only MMS or CPA groups, which represents a diminishment of cell death, as on cell viability assay. We also observed a reduction on the subpopulation with polyploidy after treatment with AVA, a fact that can be related to its antigenotoxic effect, which was the outcome observed on micronucleus assay. It is important to emphasize that there were no important changes on G1, S, and G2 phases, even after severe cell damage, and the maintenance of the cell cycle is a fundamental aspect to the reliability of micronucleus assay [32].

Iwashita et al. [33] demonstrated that pravastatin and fluvastatin reduced micronucleus formation in CHO-K1 cells after exposure to the antineoplastic bleomycin. The statins, at concentrations from 10 *μ*M to 100 *μ*M, were capable to reduce the micronucleated cell rate in pretreatment, in minor responses, and in cotreatment and posttreatment schemes being high effectives. This preventive effect was not observed in exposure to X-radiation. This corroborates with our results that demonstrated a reduction in MMS- or CPA-induced micronuclei in HepG2 cells after 6 h and 24 h of cotreatment. The earlier response of AVA against MMS is related to nitrogen heterocyclic compound capacity to reduce the reactivity of sulfonates [34] and probably the later response against CPA was due to AVA's neutralization of epoxide radicals, from CPA metabolism by CYP coenzymes [35]. So, AVA was able to act as a scavenger, protecting DNA from direct and indirect alkylation-mediated point mutations, genotoxicity, and cellular death, reducing the redox status and the genomic instability. These protective effects can avoid mitotic catastrophe [36] and are expected for a good antimutagen.

In summary, our data showed that AVA reduces the alkylation-mediated DNA damage in different *in vitro* experimental models. In a bacterial model, AVA was more effective to prevent direct than indirect damage in TA1535 (cotreatment) and TA100 (pretreatment). Cytoprotection of AVA at low doses (0.1–10.0 *μ*M) was observed after 24 h of cotreatment with MMS or CPA at their LC_50_, causing an increase in HepG2 survival rates. AVA had decrease effect in AA-induced micronucleus formation and cell cycle alterations in HepG2 cells.

## 5. Conclusion

This study supports the hypothesis that atorvastatin can be considered a chemopreventive agent, acting as antimutagenic, antigenotoxic, and cytoprotective compound, and permits to clarify about its mechanism of action, reducing the oxidative microenvironment, scavenging alkylating agents directly, or neutralizing their metabolites, and thus protecting specifically against DNA damages.

## Figures and Tables

**Table 1:** Effects of atorvastatin after cotreatment with alkylating agents on *Salmonella enterica typhimurium* strains TA104 and TA102.

Atorvastatin (*μ*M)		Coincubation	TA104			TA102		
			*His^+^*	MI	% reduction	*His^+^*	MI	% reduction
—	−S9	DMSO 1%	400 ± 31	1.00	—	250 ± 31	1.00	—
0	−S9	MMS (100 *μ*M)	897 ± 55	2.24	0.00	578 ± 55	2.31	0.00
20	−S9		801 ± 32	2.00	19.36	525 ± 32	2.10	16.03
100	−S9		776 ± 64	1.94	24.19	515 ± 64	2.06	19.08
200	−S9		736 ± 91	1.84	32.26^∗^	483 ± 91	1.93	29.01^∗^
1000	−S9		620 ± 28	1.55	55.65^∗^	443 ± 28	1.77	41.22^∗^
—	+S9	DMSO 1%	455 ± 41	1.00	—	280 ± 41	1.00	—
0	+S9	CPA (150 *μ*M)	1092 ± 85	2.40	0.00	588 ± 85	2.1	0.00
20	+S9		969 ± 54	2.13	19.29	566 ± 54	2.02	7.27
100	+S9		933 ± 38	2.05	25.00^∗^	560 ± 38	2.00	9.09
200	+S9		905 ± 42	1.99	29.29^∗^	543 ± 42	1.94	14.55
1000	+S9		829 ± 11	1.82	41.43^∗^	496 ± 11	1.77	30.00^∗^

MMS: methyl methanesulfonate; CPA: cyclophosphamide; *His^+^*: revertant colonies; MI: mutagenicity index. ^∗^*p* < 0.01 versus only MMS or only CPA (one-way ANOVA followed by a Dunnett's post hoc test).

**Table 2:** Effects of atorvastatin after cotreatment with alkylating agents on *Salmonella enterica typhimurium* strains TA1535 and TA100.

Atorvastatin (*μ*M)		Cotreatment	TA1535		TA100		
			*His^+^*	MI	% reduction	*His^+^*	MI	% reduction
—	−S9	DMSO 1%	25 ± 2	1.00	—	100 ± 5	1.00	—
0	−S9	MMS (100 *μ*M)	71 ± 5	2.84	0.00	212 ± 11	2.14	0.00
20	−S9		50 ± 4	2.00	45.65^∗^	204 ± 18	2.04	7.14
100	−S9		40 ± 5	1.60	67.39^∗^	198 ± 22	1.98	12.5
200	−S9		31 ± 2	1.24	86.95^∗^	190 ± 13	1.90	19.64
1000	−S9		29 ± 3	1.16	91.30^∗^	186 ± 14	1.86	23.21
—	+S9	DMSO 1%	20 ± 3	1.00	—	112 ± 9	1.00	—
0	+S9	CPA (150 *μ*M)	56 ± 6	2.80	0.00	239 ± 30	2.13	0.00
20	+S9		53 ± 3	2.63	9.44	235 ± 22	2.10	2.65
100	+S9		52 ± 7	2.60	11.11	230 ± 31	2.06	6.19
200	+S9		45 ± 4	2.25	30.56^∗^	224 ± 15	2.00	11.50
1000	+S9		41 ± 8	2.04	42.22^∗^	216 ± 25	1.93	17.70

MMS: methyl methanesulfonate; CPA: cyclophosphamide; *His^+^*: revertant colonies; MI: mutagenicity index. ^∗^*p* < 0.01 versus only MMS or only CPA (one-way ANOVA followed by a Dunnett's post hoc test).

**Table 3:** Effects of atorvastatin after pretreatment and posttreatment with alkylating agents on *Salmonella enterica typhimurium* strain TA100.

Atorvastatin (*μ*M)			TA100					
			Pretreatment			Posttreatment		
			*His^+^*	MI	% reduction	*His^+^*	MI	% reduction
—	−S9	DMSO 1%	102 ± 17	1.00		127 ± 4	1.00	—
0	−S9	MMS (100 *μ*M)	230 ± 23	2.26	0	264 ± 31	2.09	0
20	−S9		171 ± 14	1.68	45.97^∗^	257 ± 26	2.03	5.69
100	−S9		117 ± 2	1.14	88.57^∗^	248 ± 23	1.96	11.86
200	−S9		113 ± 6	1.11	91.43^∗^	237 ± 22	1.87	20.10
1000	−S9		103 ± 4	1.02	98.7^∗^	233 ± 26	1.84	23.00
—	+S9	DMSO 1%	100 ± 16	1.00	—	105 ± 1	1.00	—
0	+S9	CPA (150 *μ*M)	244 ± 8	2.44	0	278 ± 33	2.66	0.00
20	+S9		130 ± 30	1.30	79.4^∗^	257 ± 5	2.46	12.04
100	+S9		127 ± 19	1.27	81.6^∗^	227 ± 3	2.37	17.44
200	+S9		111 ± 9	1.11	92.59^∗^	216 ± 8	2.34	18.49
1000	+S9		103 ± 2	1.03	97.92^∗^	207 ± 23	2.30	21.48

MMS: methyl methanesulfonate; CPA: cyclophosphamide; *His^+^*: revertant colonies; MI: mutagenicity index. ^∗^*p* < 0.01 versus only MMS or only CPA (one-way ANOVA followed by a Dunnett's post hoc test).

**Table 4:** HepG2 cytotoxicity of compounds after 24 h of exposure.

Compound	LC_50_ (*μ*M)
AVA	>1000
MMS	18.67 ± 6.67
CPA	98.71 ± 11.50

LC_50_: lethal concentration of 50%; MMS: methyl methanesulfonate; CPA: cyclophosphamide; AVA: atorvastatin.
